# Comparing CPU and GPU compute of PERMANOVA on MI300A

**Published:** 2025-05-07

**Authors:** Igor Sfiligoi

**Affiliations:** University of California San Diego, San Diego Supercomputer Center, La Jolla, CA 92093, USA

**Keywords:** gpu, memory, permanova, benchmarking, apu

## Abstract

Comparing the tradeoffs of CPU and GPU compute for memory-heavy algorithms is often challenging, due to the drastically different memory subsystems on host CPUs and discrete GPUs. The AMD MI300A is an exception, since it sports both CPU and GPU cores in a single package, all backed by the same type of HBM memory. In this paper we analyze the performance of Permutational Multivariate Analysis of Variance (PERMANOVA), a non-parametric method that tests whether two or more groups of objects are significantly different based on a categorical factor. This method is memory-bound and has been recently optimized for CPU cache locality. Our tests show that GPU cores on the MI300A prefer the brute force approach instead, significantly outperforming the CPU-based implementation. The significant benefit of Simultaneous Multithreading (SMT) was also a pleasant surprise.

## 1 INTRODUCTION

The Permutational Multivariate Analysis of Variance (PERMANOVA) [1–2] is a popular method used in the microbiome field. It is is a non-parametric method that tests whether two or more groups of objects (e.g., samples) are significantly different based on a categorical factor, taking a distance matrix as its input. The statistical significance is assessed via a permutation test, with the assignment of objects to groups randomly permuted a number of times. This makes is extremely memory-intensive. The reference implementation is available in *scikit-bio* [3], with a further optimized version available in *unifrac-binaries* [4].

The AMD MI300A [5] is the first data-center Accelerated Processor Unit (APU) publicly available, and has been installed in several High-Performance Computing (HPC) centers. Unlike other compute setups, the MI300A combines both Central Processing Units (CPU) and Graphics Processing Units (GPU) in a single package, all of them paired with on-chip High Bandwidth Memory (HBM). This uniformity makes it a great target for comparing memory-heavy algorithmic performance between CPU and GPU implementations.

## 2 PERMANOVA INNER ALGORITHM

This section describes the most time-consuming part of the PERMANOVA algorithm, the parallel pseudo-F partial statistics computation (*permanova_f_stat_sW*). While the actual PERMANOVA includes several other steps that happen before and after, they add minimal overhead and will thus be ignored in this paper. The interested reader can find the complete source code at [6].

A typical PERMANOVA invocation uses a distance matrix between 1k^2^ and 100k^2^ elements, and computes the pseudo- F partial statistic on between 1k and 1M permutations. Each permutation is independent, making that dimension the most obvious parallelization target. The original, brute force implementation of PERMANOVA’s parallel pseudo-F partial statistic is presented in Algorithm 1.

**ALGORITHM 1:** Original brute force permanova_f_stat_sW

float **permanova_f_stat_sW_one**(mat[], n_dims, grouping[], inv_group_sizes[]) {
s_W = 0.0;
for (int row=0; row < (n_dims-1); row++) { // no columns in last row
for (int col=row+1; col < n_dims; col++) { // diagonal is always zero
int group_idx = grouping[row];
if (grouping[col] == group_idx) {
const float * mat_row = mat + uint64_t(row)*uint64_t(n_dims);
val = mat_row[col];
s_W += val * val * inv_group_sizes[group_idx];;
}
}}
return s_W;
}
void **permanova_f_stat_sW_T**(mat[], n_dims, groupings[], n_perms, inv_group_sizes[], group_sWs[]) {
#pragma omp parallel for
for (int p=0; p < n_perms; p++) {
group_sWs[p] = permanova_f_stat_sW_one(mat, n_dims, groupings + p*n_dims, inv_group_sizes);
}

A careful look at the logic shows that the *grouping* array is accessed in a tiled manner, so the obvious step on CPU cores was to implement a tiled version of the algorithm. Unfortunately, many compilers do not support, or properly implement, the OpenMP *tile* directive on non-square nested loops, so we had to explicitly split the two loops by hand. As a side effect, we discovered that we could reuse the access to *inv_group_sizes* array in the innermost loop, saving both memory accesses and compute. The tiled version is available as Algorithm 2.

**ALGORITHM 2:** Tiled permanova_f_stat_sW

float **permanova_f_stat_sW_one**(mat[], n_dims, grouping[], inv_group_sizes[]) {
s_W = 0.0;
for (int trow=0; trow < (n_dims-1); trow+=TILE) { // no columns in last row
for (int tcol=trow+1; tcol < n_dims; tcol+=TILE) { // diagonal is always zero
for (uint32_t row=trow; row < min(trow+TILE,n_dims-1); row++) {
min_col = std::max(tcol,row+1); max_col = std::min(tcol+TILE,n_dims);
float * mat_row = mat + row*n_dims;
int group_idx = grouping[row];
local_s_W = 0.0;
for (uint32_t col=min_col; col < max_col; col++) {
if (grouping[col] == group_idx) {
val = mat_row[col];
local_s_W += val * val;
}
}
s_W += local_s_W*inv_group_sizes[group_idx];
}}}
return s_W;
}
void **permanova_f_stat_sW_T**(mat[], n_dims, groupings[], n_perms, inv_group_sizes[], group_sWs[]) {
#pragma omp parallel for
for (int p=0; p < n_perms; p++) {
group_sWs[p] = permanova_f_stat_sW_one(mat, n_dims, groupings + p*n_dims, inv_group_sizes);
}

When it came time to port the code to GPU compute, we chose to use the GPU-offloading syntax of OpenMP [7], which required the insertion of only two pragma lines. Due to the much higher parallel nature of the GPUs, we parallelize both at permutation and distance matrix level there, as shown in Algorithm 3. Note that we ended up using the brute force version of the algorithm, as any attempt to tile the algorithm resulted in drastically slower execution.

**ALGORITHM 3:** GPU implementation of the brute force permanova_f_stat_sW

float **permanova_f_stat_sW_one**(mat[], n_dims, grouping[], inv_group_sizes[]) {
s_W = 0.0;
#pragma omp parallel for collapse(2) reduction(+:s_W)
for (int row=0; row < (n_dims-1); row++) { // no columns in last row
for (int col=row+1; col < n_dims; col++) { // diagonal is always zero
int group_idx = grouping[row];
if (grouping[col] == group_idx) {
const float * mat_row = mat + uint64_t(row)*uint64_t(n_dims);
val = mat_row[col];
s_W += val * val * inv_group_sizes[group_idx];;
}
}}
return s_W;
}
void **permanova_f_stat_sW_T**(mat[], n_dims, groupings[], n_perms, inv_group_sizes[], group_sWs[]) {
#pragma omp target teams distribute
for (int p=0; p < n_perms; p++) {
group_sWs[p] = permanova_f_stat_sW_T_one(mat, n_dims, groupings + p*n_dims, inv_group_sizes);
}

## 3 BENCHMARK RESULTS ON MI300A

The above algorithms have been benchmarked as part of the early-user phase of the SDSC Cosmos system. Each Cosmos node contains 4 MI300A, but we used only one at a time, to contain the memory traffic within the single chip. The CPU code was compiled using gcc 14.2, while the GPU code was compiled using the clang-based AOMP 20.0 [8].

**Figure 1:**
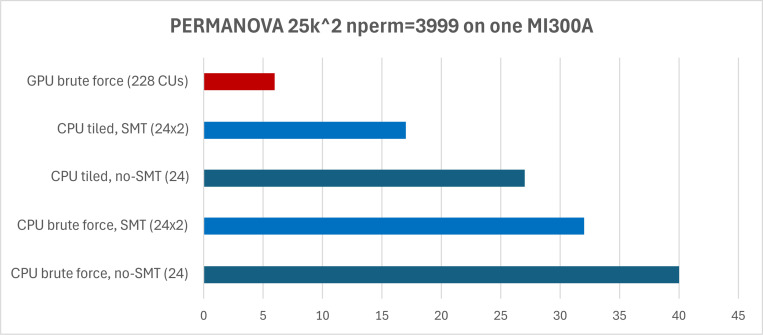
PERMANOVA execution time by algorithm and resource. The horizontal axis is expressed in seconds (lower is better).

The input distance matrix had a size of 25145^2^ and was the result of computing the Unweighted Unifrac [9] on the Earth Microbiome Project (EMP) data. The number of permutations was 3999; the magnitude was chosen to be both large enough to exploit the GPU parallelism, small enough to result in reasonable execution time and would represent a realistic use case. The summary results for both CPU and GPU execution are available in Figure 1.

As can be seen, using the GPU compute units indeed results is drastically faster execution time. Compared to the same brute force algorithm on a non-SMT CPU setup, the GPU implementation is over 6x faster. That said, the more flexible nature of the CPU compute units allows for smarter algorithms, which claw back some of that advantage. This is especially noticeable when paired with Simultaneous Multithreading (SMT), that exposes two hardware threads for each physical CPU core.

In summary, even with an identical memory subsystem attached to both CPU and GPU compute units, massively parallel but memory-intensive algorithms like PERMANOVA do benefit from GPU compute. Some of the CPU-focused optimizations may however not directly translate to the GPU implementations, thus likely requiring some device-specific code.

## Supplementary Material

Supplement 1

## CCS CONCEPTS

- Computing methodologies~Parallel computing methodologies
