# Supplementary material for: Comparing CPU and GPU compute of PERMANOVA on MI300A
Source: ArXiv. 2025 May 7:arXiv:2505.04556v1. Preprint. [Version 1] (PMC12083706)
Supplement: Supplement 1 [file NIHPP2505.04556v1-supplement-1.pdf]

APPENDIX

A1 Hardware details

Each Cosmos node contains 4 MI300A APUs. The tested nodes was in SPX mode, the user thus sees 192 logical cores and 4 logical GPUs, as outlined below:

```
> lscpu

Architecture:             x86_64
  CPU op-mode(s):         32-bit, 64-bit
  Address sizes:          48 bits physical, 48 bits virtual
  Byte Order:             Little Endian
CPU(s):                   192
  On-line CPU(s) list:    0-191
Vendor ID:                AuthenticAMD
  Model name:             AMD Instinct MI300A Accelerator
    CPU family:           25
    Model:                144
    Thread(s) per core:   2
    Core(s) per socket:   24
    Socket(s):            4
    Stepping:             1
    Frequency boost:      enabled
    CPU max MHz:          3700.0000
    CPU min MHz:          1500.0000
    BogoMIPS:             7400.37
…
Caches (sum of all):
  L1d:                    3 MiB (96 instances)
  L1i:                    3 MiB (96 instances)
  L2:                     96 MiB (96 instances)
  L3:                     384 MiB (12 instances)
NUMA:
  NUMA node(s):           4
  NUMA node0 CPU(s):      0-23,96-119
  NUMA node1 CPU(s):      24-47,120-143
  NUMA node2 CPU(s):      48-71,144-167
  NUMA node3 CPU(s):      72-95,168-191
…
> rocm-smi --showtoponuma

============================ ROCm System Management Interface ============================
======================================== Numa Nodes ======================================
GPU[0]         : (Topology) Numa Node: 0
GPU[0]         : (Topology) Numa Affinity: 0
GPU[1]         : (Topology) Numa Node: 1
GPU[1]         : (Topology) Numa Affinity: 1
GPU[2]         : (Topology) Numa Node: 2
GPU[2]         : (Topology) Numa Affinity: 2
GPU[3]         : (Topology) Numa Node: 3
GPU[3]         : (Topology) Numa Affinity: 3
================================== End of ROCm SMI Log ===================================
```

Only one APU was used during the tests, by setting

```
> export ROCR_VISIBLE_DEVICES=0
> taskset -c 0-23,96-119 <command>
```

A2 Memory subsystem benchmarks

The theoretical peak performance of each MI300A APU is 5.3 TB/s, as per AMD data sheet: https://www.amd.com/content/dam/amd/en/documents/instinct-tech-docs/data-sheets/amd-instinct-mi300a-data-sheet.pdf

The achievable memory throughput using a GPU-aware variant of the STREAM benchmark. The source code of modified STREAM benchmark test is available at https://github.com/sfiligoi/STREAM-OMPGPU . As can be seen, the GPU cores report approximately 3.0 TB/s, while the CPU cores report approximately 0.2 TB/s achievable memory throughput in the Triad test.

```
> export ROCR_VISIBLE_DEVICES=0
> taskset -c 0-23,96-119 ./stream.large.exe
-------------------------------------------------------------
STREAM version $Revision: 5.10 $
-------------------------------------------------------------
This system uses 8 bytes per array element.
-------------------------------------------------------------
Array size = 1000000000 (elements), Offset = 0 (elements)
Memory per array = 7629.4 MiB (= 7.5 GiB).
Total memory required = 22888.2 MiB (= 22.4 GiB).
Each kernel will be executed 10 times.
 The *best* time for each kernel (excluding the first iteration)
 will be used to compute the reported bandwidth.
-------------------------------------------------------------
Number of Threads requested = 48
Number of Threads counted = 48
-------------------------------------------------------------
Your clock granularity/precision appears to be 1 microseconds.
Each test below will take on the order of 51275 microseconds.
   (= 51275 clock ticks)
Increase the size of the arrays if this shows that
you are not getting at least 20 clock ticks per test.
-------------------------------------------------------------
WARNING -- The above is only a rough guideline.
For best results, please be sure you know the
precision of your system timer.
-------------------------------------------------------------
Function    Best Rate MB/s  Avg time     Min time     Max time
Copy:          199503.7     0.081749     0.080199     0.089379
Scale:         198570.4     0.080648     0.080576     0.080715
Add:           209086.6     0.116079     0.114785     0.120439
Triad:         209123.1     0.117878     0.114765     0.120415
-------------------------------------------------------------
Solution Validates: avg error less than 1.000000e-13 on all three arrays
-------------------------------------------------------------
```

```
> export ROCR_VISIBLE_DEVICES=0
> export HSA_XNACK=1
> taskset -c 0-23,96-119 ./stream.amd_apu.exe
-------------------------------------------------------------
STREAM version $Revision: 5.10 $
-------------------------------------------------------------
This system uses 8 bytes per array element.
-------------------------------------------------------------
Array size = 1000000000 (elements), Offset = 0 (elements)
Memory per array = 7629.4 MiB (= 7.5 GiB).
Total memory required = 22888.2 MiB (= 22.4 GiB).
Each kernel will be executed 10 times.
 The *best* time for each kernel (excluding the first iteration)
 will be used to compute the reported bandwidth.
-------------------------------------------------------------
Using accelerator
-------------------------------------------------------------
Your clock granularity/precision appears to be 1 microseconds.
Each test below will take on the order of 5797 microseconds.
   (= 5797 clock ticks)
Increase the size of the arrays if this shows that
you are not getting at least 20 clock ticks per test.
-------------------------------------------------------------
WARNING -- The above is only a rough guideline.
For best results, please be sure you know the
precision of your system timer.
-------------------------------------------------------------
Function    Best Rate MB/s  Avg time     Min time     Max time
Copy:         2981158.7     0.005496     0.005367     0.005596
Scale:        3056376.7     0.005385     0.005235     0.005466
Add:          3188574.5     0.007736     0.007527     0.007934
Triad:        3160344.6     0.007783     0.007594     0.007941
-------------------------------------------------------------
Solution Validates: avg error less than 1.000000e-13 on all three arrays
-------------------------------------------------------------
```
